# Supplementary material for: No detectable Weddell Sea Antarctic Bottom Water export during the Last and Penultimate Glacial Maximum
Source: Nat Commun. 2020 Jan 22;11:424. doi: 10.1038/s41467-020-14302-3 (PMC6976697; doi:10.1038/s41467-020-14302-3)
Supplement: Supplementary file 1 — Supplementary Information [file 41467_2020_14302_MOESM1_ESM.pdf]

Supplementary Information for

# No detectable Weddell Sea Antarctic Bottom Water export during the Last and Penultimate Glacial Maximum

by

Huang et al.

**Supplementary Discussion.**

**Potential impact of Ice Rafted Debris (IRD) on Pb and Nd isotope composition.** Previous studies suggested that Ice Rafted Debris (IRD) may have a substantial impact on both Pb and Nd isotope compositions in high latitude oceans and authigenic phases formed in the sediments during elevated IRD flux in the water column[1, 2, 3, 4]. Since ODP Site 1094 and core PS1768-8 were recovered in the flow path of Antarctic Iceberg Alley[5], the temporally elevated IRD input may in theory compromise our results. The locations of ODP Site 1094 and core PS1768-8 are very close to each other, for which reason we only compare IRD contents from PS1768-8 to our $^{206}Pb/^{204}Pb$ and $\varepsilon_{Nd}$ results. As shown in Extended Data Figure S4, strong IRD flux was only recorded during glacial times, but in these cold periods our $^{206}Pb/^{204}Pb$ evolution is remarkably smooth, arguing against a significant impact of IRD-sourced Pb on our extracted signal[2, 3]. As shown in Supplementary Figure 3 and 4, the variable IRD concentrations only bear a weak correlation with extracted $\varepsilon_{Nd}$ records both in core PS1599-3[6] and PS1768-8[7]. In particular, the $\varepsilon_{Nd}$ is almost invariant while the IRD content peaked in various sections throughout the cores. Strikingly, sedimentary sections featuring the major transitions in $\varepsilon_{Nd}$ found in our record late during Termination I and II do not contain significant amounts of IRD. We therefore suggest that our $\varepsilon_{Nd}$ records are not influenced by IRD-sourced Nd either.

**Anthropogenic Pb contamination.** Given that the oceanic Pb isotope signal today is largely controlled by anthropogenically sourced Pb[8, 9], the modern seawater Pb isotope compositions do not reflect natural Pb isotope signatures of different water masses in the past. To circumvent this problem, we extracted the pre-industrial seawater Pb isotope signature from the authigenic Fe-Mn oxyhydroxide fraction in 20 individual core-top sediment samples in various parts of the Weddell Sea, Drake Passage and Atlantic sector of the Southern Ocean further north (Supplementary Data Table 1). Since Fe-Mn nodules are the most reliable Pb isotope archive available, we compare our Pb isotope records with previous Pb isotope datasets based on nearby surface Fe-Mn nodules[10] (Supplementary Figure 5). Any modern anthropogenic Pb contamination would be detectable via its extremely unradiogenic (low) Pb isotopic signature[11, 12]. As shown in Supplementary Figure 5, Pb isotope compositions extracted from sediments are systematically more radiogenic (higher) than the Fe-Mn nodule Pb isotope signal, proving strong support that anthropogenic Pb contamination is not controlling our sedimentary Pb isotope records.

**Dust influence on Pb isotope compositions.** Previous studies found that dust input in the ocean

can dominate the seawater Pb isotope signal in the past[11]. In order to determine the dust influence, we compare the dust record in EPICA Dome C[13, 14] with our Pb isotope record (Supplementary Figure 6). Most intervals covered within our Pb record correspond to times of insignificant dust deposition. Only the Glacial Maxima were marked by strong dust input. Previously published dust Pb isotope signatures were characterized by frequent and large-amplitude fluctuations between radiogenic and unradiogenic Pb isotope signatures[14]. Our seawater Pb isotope record extracted from bulk sediment does not show any correlation with either dust intensity or dust Pb isotopic signatures in the ice core. This suggests that our Pb isotope record is not offset by dust-derived Pb.

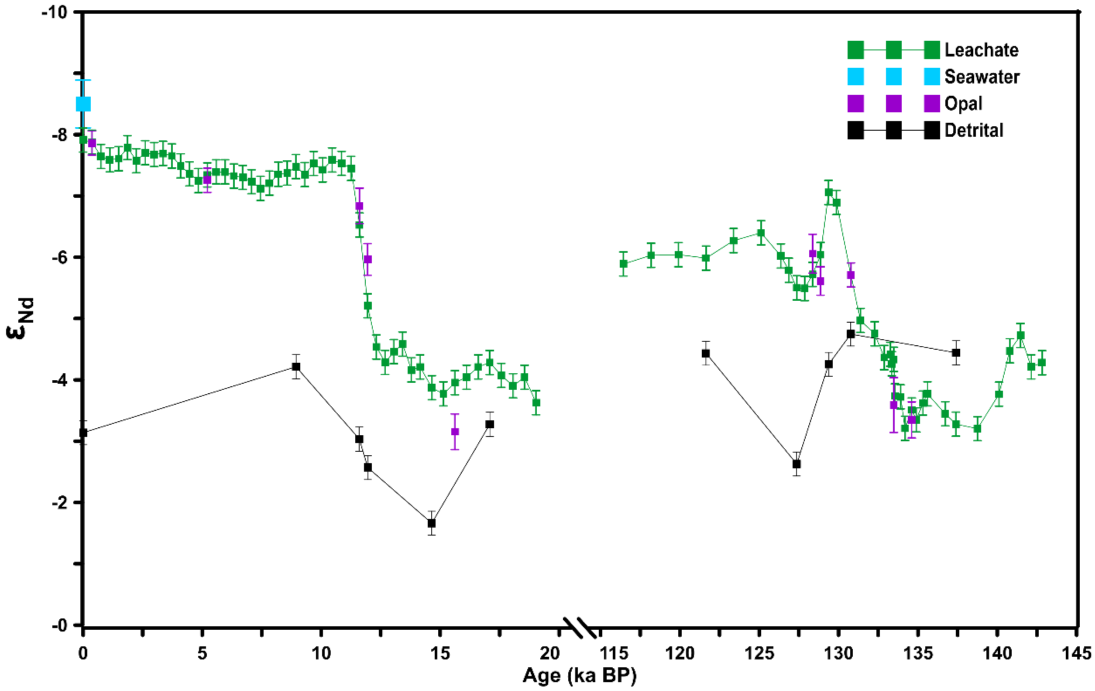


**Supplementary Figure 1:** Extracted authigenic $\varepsilon_{Nd}$ in core PS1768-8 compared with $\varepsilon_{Nd}$ in the actual seawater, from opal and detrital fraction. The seawater $\varepsilon_{Nd}$ is taken from the nearest seawater sampling station (Station 113, 53.6°S, 2.0°E, water depth 2400 m)[15]. Error bars correspond to the 2σ external error of the $\varepsilon_{Nd}$ measurements.

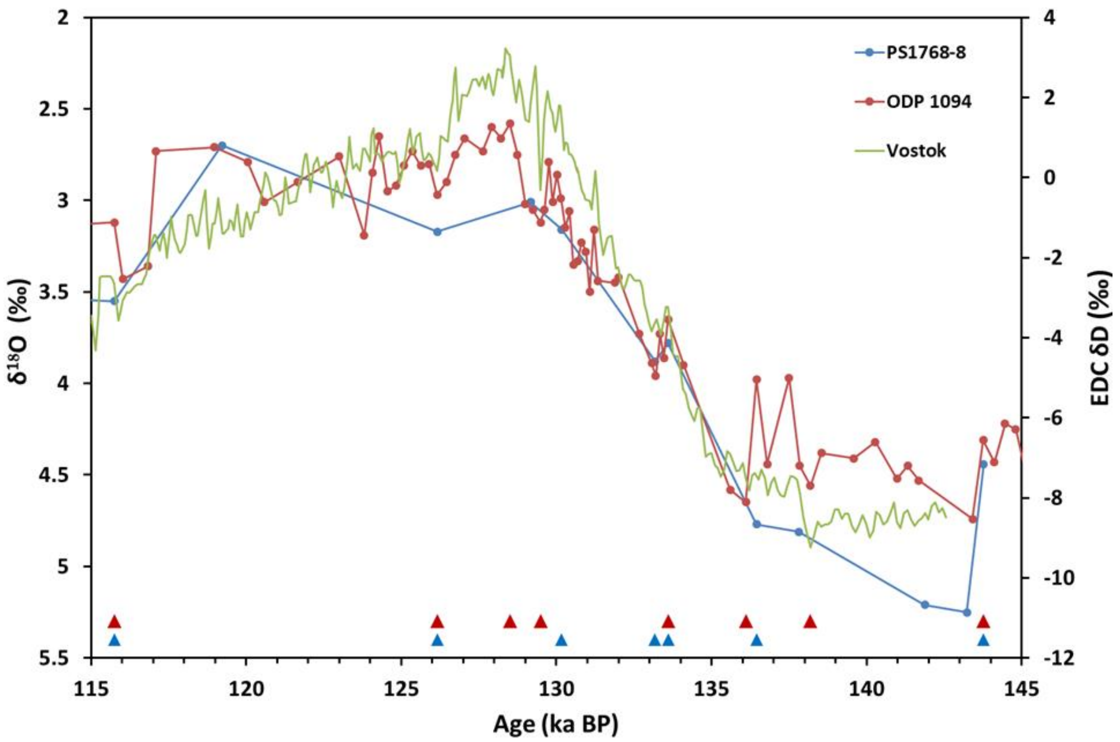


**Supplementary Figure 2:** Age model found for Termination II from fine-tuning the composite planktonic $\delta^{18}O$ record[16, 17] to Vostok $\delta D$[18] . The triangles represent the tie-points defined to tune records.

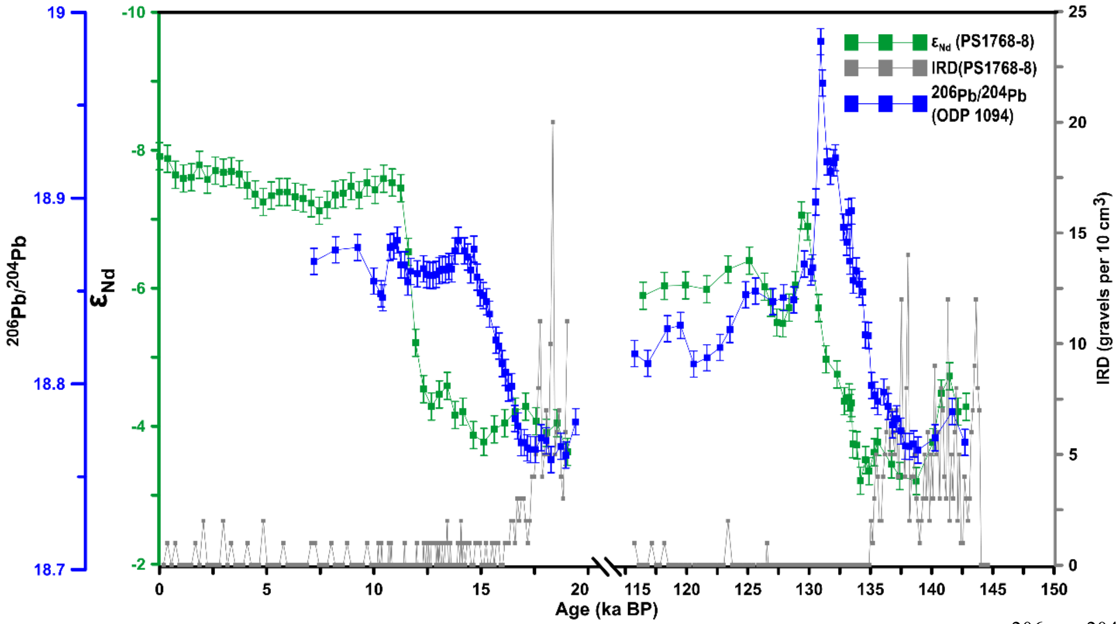


**Supplementary Figure 3:** Extracted authigenic Fe-Mn oxyhydroxide derived $^{206}Pb/^{204}Pb$ at ODP Site 1094 and $\varepsilon_{Nd}$ in core PS1768-8 compared with its IRD concentrations[7]. Error bars correspond to the 2σ external error of the $^{206}Pb/^{204}Pb$ and $\varepsilon_{Nd}$ measurements. Major Pb and Nd isotopic changes did not coincide with changes in IRD concentration during Terminations I and II.

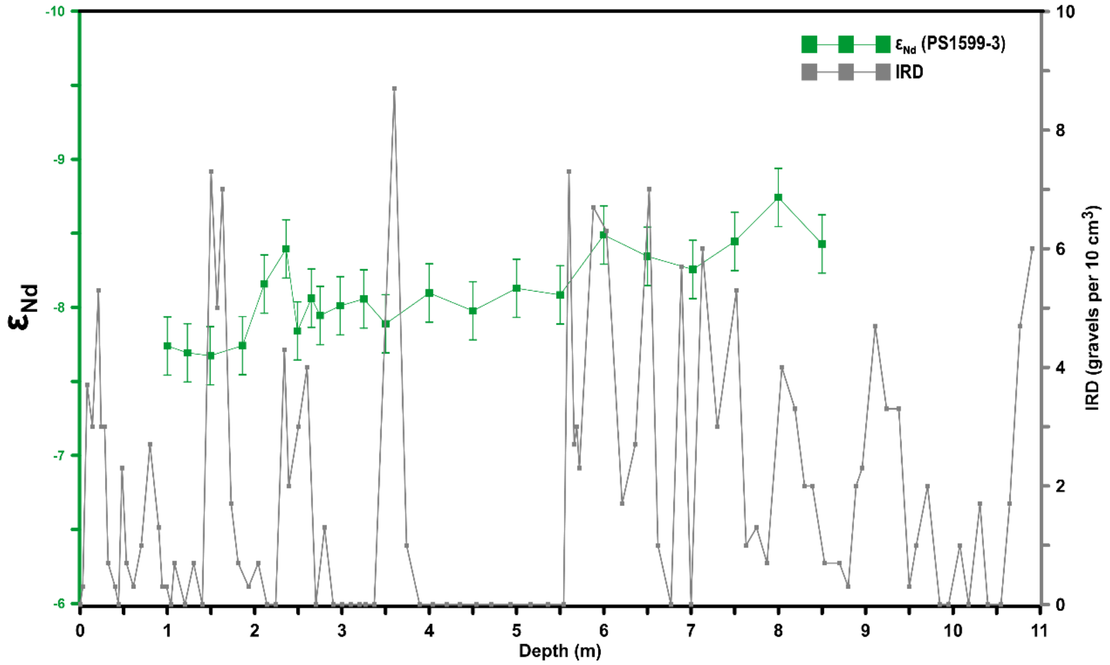


**Supplementary Figure 4:** Extracted authigenic $\varepsilon_{Nd}$ in core PS1599-3 compared with IRD concentrations in the core[6]. Error bars correspond to the 2σ external error of the $\varepsilon_{Nd}$ measurements. Covariation between Nd isotopic changes and IRD concentration changes are not apparent.

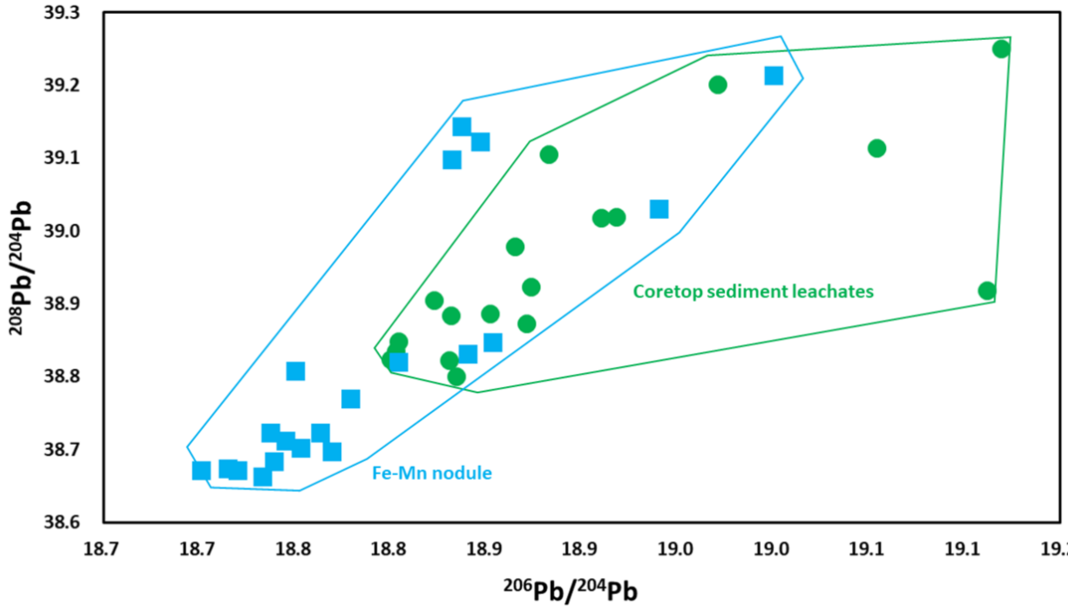


**Supplementary Figure 5:** Comparison of Pb isotopic compositions between surface Fe-Mn nodules[10] and core-top sediment leachates in the Weddell Sea, Drake Passage and Atlantic sector of the Southern Ocean. Pb isotope compositions of core-top sediment leachates are more radiogenic (higher) than the Fe-Mn nodule Pb isotope signal, suggesting that anthropogenic Pb contamination, which is unradiogenic (normally $^{206}Pb/^{204}Pb<18.7$)[11], does not dominate our sedimentary Pb isotope records.

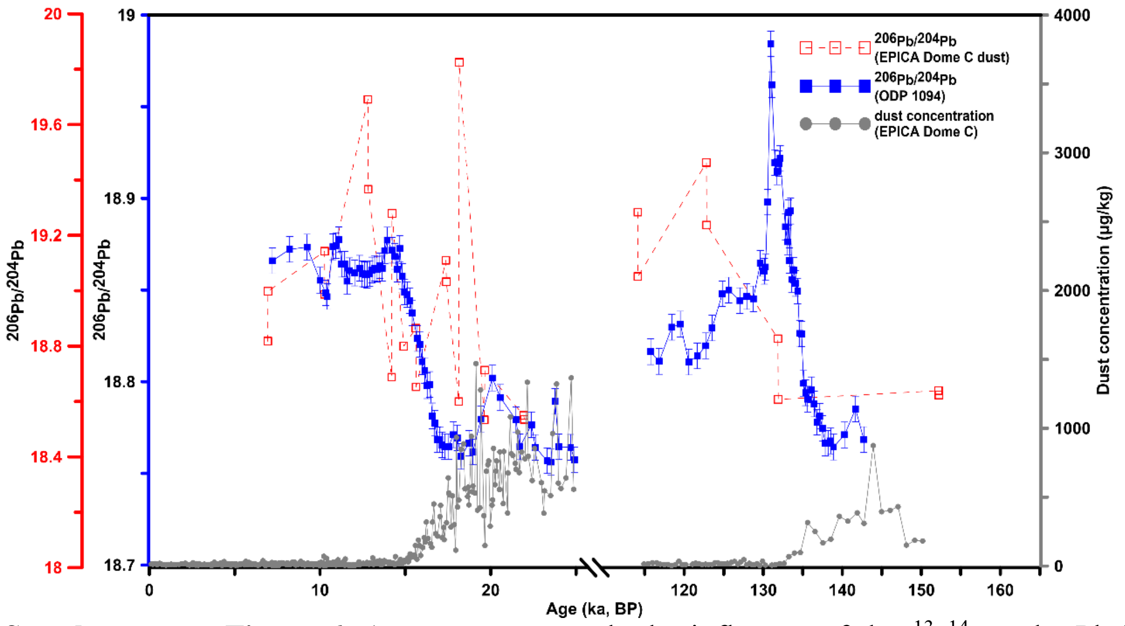


**Supplementary Figure 6:** Assessment towards the influence of dust[13, 14] on the Pb isotope composition at ODP Site 1094. Error bars correspond to the 2σ external error of the $^{206}Pb/^{204}Pb$ and $^{208}Pb/^{204}Pb$. Our $^{206}Pb/^{204}Pb$ record neither co-vary with dust concentrations, nor dust Pb isotopic signatures during Terminations I and II.

**Supplementary References**


1. Blaser P*, et al.* The resilience and sensitivity of Northeast Atlantic deep water εNd to overprinting by detrital fluxes over the past 30,000 years. *Geochimica et Cosmochimica Acta* **245**, 79-97 (2019).

2. Crocket KC, Vance D, Foster GL, Richards DA, Tranter M. Continental weathering fluxes during the last glacial/interglacial cycle: insights from the marine sedimentary Pb isotope record at Orphan Knoll, NW Atlantic. *Quaternary Science Reviews* **38**, 89-99 (2012).

3. Kurzweil F, Gutjahr M, Vance D, Keigwin L. Authigenic Pb isotopes from the Laurentian Fan: Changes in chemical weathering and patterns of North American freshwater runoff during the last deglaciation. *Earth and Planetary Science Letters* **299**, 458-465 (2010).

4. Roberts NL, Piotrowski AM. Radiogenic Nd isotope labeling of the northern NE Atlantic during MIS 2. *Earth and Planetary Science Letters* **423**, 125-133 (2015).

5. Weber ME*, et al.* Millennial-scale variability in Antarctic ice-sheet discharge during the last deglaciation. *Nature* **510**, 134-138 (2014).

6. Michels K*, et al.* Grain size composition of sediment cores from the Weddell Sea, Antarctica. Pangaea http://dx.doi.org/10.1594/PANGAEA.472241 (2002).

7. Diekmann B*, et al.* Ice rafted debris distribution in 16 sediment cores from the South Atlantic. Pangaea http://dx.doi.org/10.1594/PANGAEA.732965 (2003).

8. Flegal AR, Maring H, Niemeyer S. Anthropogenic lead in Antarctic sea water. *Nature* **365**, 242-244 (1993).

9. Lee J-M, Boyle EA, Gamo T, Obata H, Norisuye K, Echegoyen Y. Impact of anthropogenic Pb and ocean circulation on the recent distribution of Pb isotopes in the Indian Ocean. *Geochimica et Cosmochimica Acta* **170**, 126-144 (2015).

10. Abouchami W, Goldstein SL. A lead isotopic study of circum-antarctic manganese nodules. *Geochimica et Cosmochimica Acta* **59**, 1809-1820 (1995).

11. Bollhöfer A, Rosman KJR. Isotopic source signatures for atmospheric lead: the Southern Hemisphere. *Geochimica et Cosmochimica Acta* **64**, 3251-3262 (2000).

12. Bollhöfer A, Rosman KJR. The temporal stability in lead isotopic signatures at selected sites in the Southern and Northern Hemispheres. *Geochimica et Cosmochimica Acta* **66**, 1375-1386 (2002).

13. Lambert F*, et al.* Dust-climate couplings over the past 800,000 years from the EPICA Dome C ice core. *Nature* **452**, 616-619 (2008).

14. Vallelonga P*, et al.* Lead isotopic compositions in the EPICA Dome C ice core and Southern Hemisphere Potential Source Areas. *Quaternary Science Reviews* **29**, 247-255 (2010).

15. Stichel T, Frank M, Rickli J, Haley BA. The hafnium and neodymium isotope composition of seawater in the Atlantic sector of the Southern Ocean. *Earth and Planetary Science Letters* **317-318**, 282-294 (2012).

16. Hodell DA, Charles CD, Curtis JH, Mortyn PG, Ninnemann US, Venz KA. Stable isotope record of benthic and planktonic foraminifera in sediment cores of ODP Leg 177, Southern Ocean. In: *Proceedings of the Ocean Drilling Program, Scientific Results, College Station, TX (Ocean Drilling Program), 177, 1-26, https://doi.org/10.2973/odp.proc.sr.177.120.2003*) (2003).

17. Niebler H-S. Stable isotope record of foraminifera from South Atlantic sediments with reconstruction of paleotemperatures and paleosalinities. Pangaea http://dx.doi.org/*10.1594/PANGAEA.835327* (1995).

18. Petit JR*, et al.* Climate and atmospheric history of the past 420,000 years from the Vostok ice core, Antarctica. *Nature* **399**, 429-436 (1999).
