# Supplementary material for: No detectable Weddell Sea Antarctic Bottom Water export during the Last and Penultimate Glacial Maximum
Source: Nat Commun. 2020 Jan 22;11:424. doi: 10.1038/s41467-020-14302-3 (PMC6976697; doi:10.1038/s41467-020-14302-3)
Supplement: Supplementary file 3 — Description of Additional Supplementary Files [file 41467_2020_14302_MOESM3_ESM.pdf]

Description of Additional Supplementary Files

File name: Supplementary Data 1

Description: Site 1094 Pb isotope results.

File name: Supplementary Data 2

Description: Coretop Pb isotope results.

File name: Supplementary Data 3

Description: ε_{Nd} (PS1768-8 and PS1599-3).
